# Computational repositioning of ethno medicine elucidated gB-gH-gL complex as novel anti herpes drug target

**DOI:** 10.1186/1472-6882-13-85

**Published:** 2013-04-15

**Authors:** Syed Hussain Basha, Deepthi Talluri, Nalini Prasad Raminni

**Affiliations:** 1 Independent Researcher, 117-D, WWSC, Guntupalli, Ibrahimpatnam, Vijayawada 521 241, India; 2 Montessori Siva sivani Institute of Science and Technology-College of Pharmacy, Mylavaram, Vijayawada, 521 230, India; 3 Department of Biotechnology, REVA Institute of Science and Management, Yelahanka, Bangalore, 560 064, India

**Keywords:** Herpes simplex virus, gB-gH-gL complex, Ethnomedicine, Docking, (3-chloro phenyl) methyl-3, 4, 5 trihydroxybenzoate

## Abstract

**Background:**

Herpes viruses are important human pathogens that can cause mild to severe lifelong infections with high morbidity. They remain latent in the host cells and can cause recurrent infections that might prove fatal. These viruses are known to infect the host cells by causing the fusion of viral and host cell membrane proteins. Fusion is achieved with the help of conserved fusion machinery components, glycoproteins gB, heterodimer gH-gL complex along with other non-conserved components. Whereas, another important glycoprotein gD without which viral entry to the cell is not possible, acts as a co-activator for the gB-gH-gL complex formation. Thus, this complex formation interface is the most promising drug target for the development of novel anti-herpes drug candidates. In the present study, we propose a model for binding of gH-gL to gB glycoprotein leading from pre to post conformational changes during gB-gH-gL complex formation and reported the key residues involved in this binding activity along with possible binding site locations. To validate the drug targetability of our proposed binding site, we have repositioned some of the most promising *in vitro*, *in vivo* validated anti-herpes molecules onto the proposed binding site of gH-gL complex in a computational approach.

**Methods:**

Hex 6.3 standalone software was used for protein-protein docking studies. Arguslab 4.0.1 and Accelrys^®^ Discovery Studio 3.1 Visualizer softwares were used for semi-flexible docking studies and visualizing the interactions respectively. Protein receptors and ethno compounds were retrieved from Protein Data Bank (PDB) and Pubchem databases respectively. Lipinski’s Filter, Osiris Property Explorer and Lazar online servers were used to check the pharmaceutical fidelity of the drug candidates.

**Results:**

Through protein-protein docking studies, it was identified that the amino acid residues VAL342, GLU347, SER349, TYR355, SER388, ASN395, HIS398 and ALA387 of gH-gL complex play an active role in its binding activity with gB. Semi flexible docking analysis of the most promising *in vitro*, *in vivo* validated anti-herpes molecules targeting the above mentioned key residues of gH-gL complex showed that all the analyzed ethno medicinal compounds have successfully docked into the proposed binding site of gH-gL glycoprotein with binding energy range between -10.4 to -6.4 K.cal./mol.

**Conclusions:**

Successful repositioning of the analyzed compounds onto the proposed binding site confirms the drug targetability of gH-gL complex. Based on the free binding energy and pharmacological properties, we propose (3-chloro phenyl) methyl-3,4,5 trihydroxybenzoate as worth a small ethno medicinal lead molecule for further development as potent anti-herpes drug candidate targeting gB-gH-gL complex formation interface.

## Background

**Figure 1:**
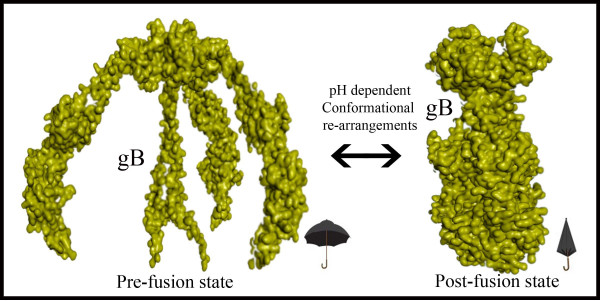
**Pictorial representation of proposed model by Heldwein, EE et.al, **[4]**for pre to post fusion conformational changes in gB.**

Almost 85% of the world population are infected with Herpes Simplex Virus type 1 and type 2 (HSV-1 and HSV-2), which are two members of Herpesviridae family [1]. HSV infects epithelial tissues primarily and then spreads to peripheral nervous system, where it establishes latency in the neurons. Recurrent HSV-1 and HSV-2 infections typically cause oral and genital lesions respectively [2]. HSV infections are more often in people with suppressed immune system like those suffering from AIDS than others [3]. These viruses are known to infect the host cells by causing the fusion of viral and host cell membrane proteins. Fusion is achieved with the help of conserved fusion machinery components that consists of glycoproteins gB, heterodimer gH-gL complex along with other non-conserved components. Formation of gB-gH-gL complex is critical for herpes viral entry into the host cell, thus making this interface the most promising drug target. During the process of this complex formation, gB was proposed to undergo dramatic conformational rearrangements (in a umbrella fashion) as shown in Figure 1 and direct binding of gH-gL was thought to be the trigger for this conformational rearrangements. Complete structural features of gB and gH-gL glycoproteins along with their proposed mode of actions can be found elsewhere in detail [4,5]. Whereas, another important glycoprotein gD, without which viral entry to the cell is not possible, acts as a co-activator for the gB-gH-gL complex formation [6,7].

### Role of ethnomedicine in finding novel antiherpes drugs

The nucleoside analog acyclovir, a widely used anti-herpes drug, is unable to cure the chronic infections and recurrence. Use of this drug often leads to the development of viral resistance coupled with side effects and viral latency [8]. Therefore discovery of novel antiherpes drugs with high specificity to inhibit the viral infection deserves great effort. On the other hand, a wide array of antimicrobial compounds with antiviral activity is described in traditional medical literature. Traditional medicine enjoys new found interest among microbiologists because of increasing antibiotic resistance and limited life span of conventional antimicrobials. It is a common practice in various fields of Biomedicine to choose plants described in traditional medicine for finding new successful therapeutic agents [9]. Most of the ethno medicinal compounds were ignored by medical practitioners despite of their proven *in vitro* and *in vivo* antiviral properties due to lack of evidence on their mode of action, effective dosage, chemical composition and toxicity [10].

Recent studies have reported promising anti Herpes viral activities of several plant extracts, some of the active constituents of this plant extracts are reported to have potential to interfere with RNA and protein synthesis, DNA replication, cellular fusion, attachment and penetration, virus entry and target cell binding, whereas some are thought to interfere in more than one step of herpes virus life cycle, resulting in complementary mechanisms of action to the existing antiviral drugs, as reviewed elsewhere in the literature [11]. In this scenario, this present work is an attempt to elucidate the possible mode of action of various promising *in vitro*, *in vivo* validated anti herpes ethno medicinal compounds to inhibit the viral fusion mechanism by attenuating the gB-gH-gL complex formation interface in a computational approach.

## Methods

### Preparation of protein receptor

The crystal structures of glycoprotein B (gB) (PDB ID: 2GUM) [4] and gH-gL complex (PDB ID: 3M1C) [5] of Herpes simplex virus, which were resolved using X-Ray diffraction method with a resolution factor of 2.10 Å and 3.00 Å respectively were obtained from Protein Data Bank (PDB) [12]. Methodology adapted for the preparation of protein receptor gH-gL complex for the semi flexible docking studies was described elsewhere in detail [13]. The modified structure so obtained was saved in .pdb format and used for all docking studies.

### Selection and preparation of ligands

Structures of the ethno medicinal compounds with proven *in vitro* and *in vivo* activity against herpes as reviewed elsewhere in literature [11] were retrieved from Pubchem database [14]. All the compounds retrieved were screened with ADME constraints according to Lipinski’s rule. Finally, 29 compounds were selected for further studies, which were in accordance with Lipinski’s rule. Preparation of the selected ligands was done using Arguslab 4.0.1 software [15], addition of missing hydrogen atoms and fidelity of all bonds was checked using “add hydrogens” and “Clean Hybridization” options respectively. Geometry optimization was done using UFF Molecular Mechanics (MM) method [16-20]. Finally, all the compounds were saved in .mol format for further docking studies.

### Protein-protein docking

Protein-protein docking was performed using Hex 6.3 software [21] between Domain II of glycoprotein gB (receptor) and H2 Domain of gH-gL heterodimer complex (ligand). Out of 2000 possible solutions, the best solution binding mode is selected to identify the key residues involved in their binding activity. Following parameters are used in Docking controls of Hex 6.3 to get the best results: Correlation type-Shape only; FFT mode-3D; Post Processing-MM minimization; Grid dimension-0.4; Solutions-2000; Receptor range-180; Step size-7.5; Ligand range-180; Step size-7.5; Twist range-360; Step size-5.5; Distance range-40; Scan step-0.8; Substeps-0; Steric scan-18; Final search-25, whereas the parameters used in clustering controls to get the best results are as follows: Max Clusters-500; Sort solutions by-Cluster; Display clusters-Best; Cluster window-200; RMS threshold-3.0; Bumps threshold-0.

### Semi-flexible docking

Docking between receptor gH-gL protein and ligand was performed using “Dock a Ligand” option of Arguslab 4.0.1 software. A spacing of 0.4 Å between the grid points was used. “ArgusDock” was selected as docking engine. Search was performed by enabling “High precision” option in Docking precision menu, “Dock” was chosen as the calculation type, “Flexible” for the ligand and “AScore” was used as the scoring function. A maximum of 150 poses were allowed to be analyzed, binding site box was set to 25 × 25 × 25 angstroms to encompass the entire active site, and each docking run was repeated three times to get the best results.

### Analysis and visualization of docking simulation results

The docking results of Arguslab 4.0.1 were saved in .pdb format. Out of 150-model results, one with the least free energy bonding data was selected. Analysis, visualization of the interactions and rendering of all the docking images was done using Accelrys^®^ Discovery Studio 3.1 Visualizer software [22].

### Pharmacological properties of the compounds

Lipinski’s Filter (http://www.scfbio-iitd.res.in/utility/LipinskiFilters.jsp), Osiris Property Explorer (http://www.organic-chemistry.org/prog/peo/) and Lazar (http://lazar.in-silico.de/predict) online servers were used to check the pharmaceutical fidelity of the drug candidates.

Molecular descriptors, such as Log P, the number of hydrogen bond donors, the number of hydrogen bond acceptors, and the molecular mass of the compounds were analyzed by utilizing Lipinski Filter. Osiris Property Explorer and Lazar were used in analyzing various attributes of the drugs, such as toxicity and drug score.

## Results and discussion

### Active site identification and validation

**Figure 2:**
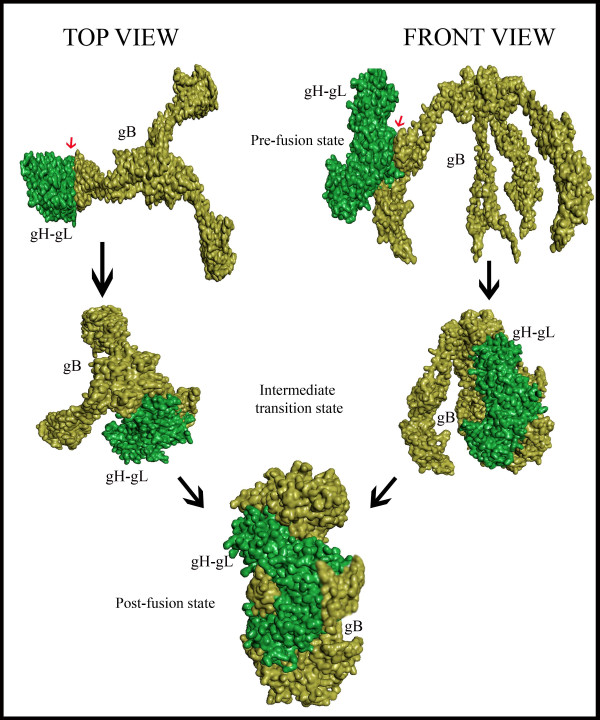
**Proposed model for binding of gH-gL (green) to gB (yellow) glycoprotein. **Left side of the picture depicts the top view of the events leading from pre to post conformational changes during gB-gH-gL complex formation, whereas the right side of the picture depicts the front view. Small red colored arrows indicate the initial proposed contact areas between these two glycoproteins.

**Figure 3:**
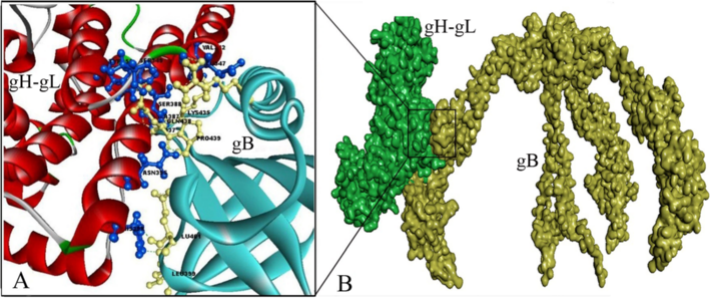
**Visualization of key residues involved in gB-gH-gL complex formation. A**) Amino acid residues involved in the gB-gH-gL complex formation, Blue colored amino acid residues VAL 342, GLU 347, SER 349, TYR 355, SER 388, ASN 395, HIS 398 and ALA 387 belong to gH-gL complex, whereas yellow colored amino acid residues LYS 435, ARG 418, GLN 438, PRO 439, LEU 399, GLU 401 and GLY 437 belong to gB glycoprotein. **B**) Hypothesized model for initial binding of gH-gL complex to gB at their H2 domain and Domain II respectively.

**Figure 4:**
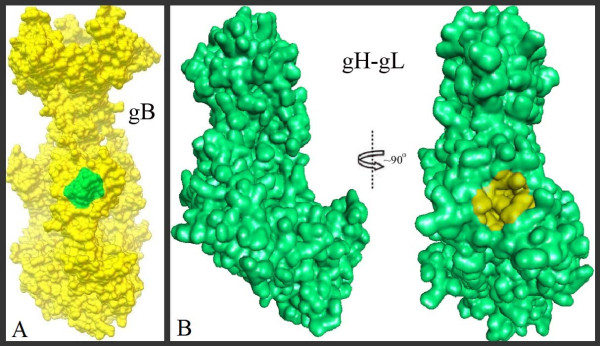
**Visualization of the glycoproteins gB and gH-gL complex: A) Proposed binding site (green) for H2 domain of gH-gL complex in gB (for better clarity binding site is shown in post conformation state of gB) B) Proposed binding site (yellow) for Domain II of gB in gH-gL complex.**

Chowdary TK et.al proposed a gB binding site in H2 domain of gH-gL complex for the gB-gH-gL complex formation through direct binding which is the key step in the formation of fusogen, subsequently leading to the viral entry into host cells [5]. Doina Atanasiu et.al, postulated that Domain II of gB contains the residues that are directly involved in gB binding to gH-gL [23]. Based on the postulates mentioned above and keeping in view of the proposed umbrella fashioned dramatic conformational rearrangements in gB glycoprotein, which was thought to be triggered by direct binding of gH-gL, lead author of this manuscript Syed Hussain Basha has hypothesized a model for pre to post conformational changes during gB-gH-gL complex formation as shown in Figure 2, and performed a protein-protein docking study between Domain II of gB and H2 domain of gH-gL complex using Hex 6.3 software to identify the key amino acid residues involved in initial contact between these two glycoproteins leading to their complex formation, and visualized that VAL 342, GLU 347, SER 349, TYR 355, SER 388, ASN 395, HIS 398 and ALA 387 amino acid residues of gH-gL complex forming hydrogen bonds with LYS 435, ARG 418, GLN 438, PRO 439, LEU 399, GLU 401 and GLY 437 of gB glycoprotein (Figure 3). The proposed binding sites of gB and gH-gL glycoproteins has been shown in Figure 4. For the present study, proposed binding site of gH-gL (Figure 4B) with amino acid residues VAL 342, GLU 347, SER 349, TYR 355, SER 388, ASN 395, HIS 398 and ALA 387 was defined as the binding site for the docking studies. To validate the drug targetability of our proposed active binding site in gH-gL, we have repositioned some of the most promising *in vitro*, *in vivo* validated anti-herpes molecules onto the proposed binding site of gH-gL complex in a computational docking simulation approach. However, further validation of proposed binding site in gH-gL as much promising drug target over proposed binding site in gB has been deduced elsewhere in detail [24].

### Interaction of the ethno compounds with active site

**Figure 5:**
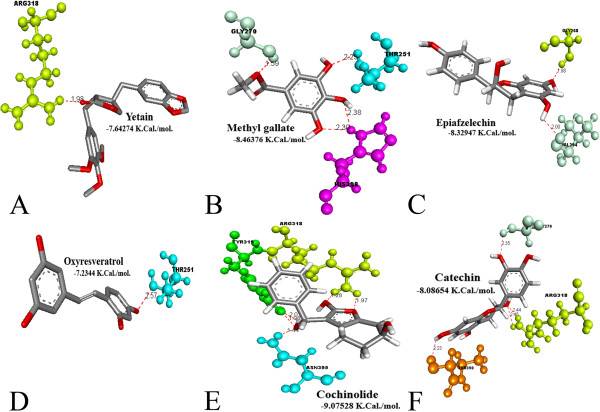
**Results of docking simulations. **Interactions between: **A**) Yatein and amino acid residue ARG 318 of gH-gL complex **B**) methylgallate and amino acid residues GLY 270, THR 251 and HIS 398 of gH-gL complex **C**) Epiafzelechin and amino acid residues GLY 268 and VAL 394 of gH-gL complex **D**) Oxyresveratrol and amino acid residue THR 251 of gH-gL complex **E**) Cochinolide and amino acid residues TYR319, ARG318 and ASN395 of gH-gL complex **F**) Catechin with the amino acid residues GLY270, ARG318 and THR350 of gH-gL complex.

**Figure 6:**
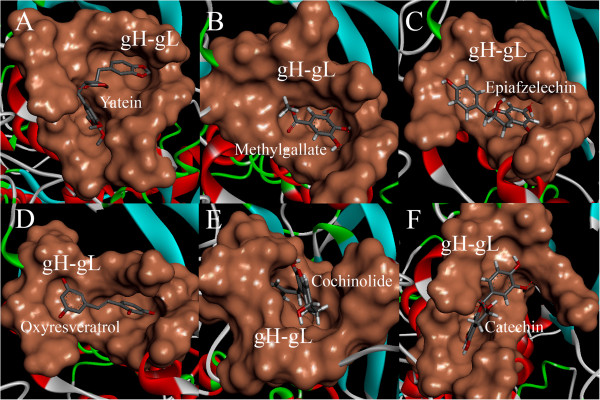
**Surface area of ligand-receptor interactions: binding of A) Yatein B) methylgallate C) Epiafzelechin D) Oxyresveratrol E) Cochinolide F) Catechin into the proposed binding site of gH-gL complex.**

The docking results in pdb were visualized using Accelrys^®^ Discovery Studio 3.1 Visualizer software. A total of 150 poses were visualized for each of the 29 ethno medicinal compounds to identify the model with best docking values, based on the binding energy interactions. The docking result showed that all the 29 compounds analyzed form hydrogen bonds with various amino acid residues in the target molecule, in the range of 0–8 hydrogen bonds (Table 1). Apart from hydrogen bonds, several other interactions like pi-cationic, pi-pi interactions and other non-covalent bonds have thought to play key role in stabilizing the docked compounds in the proposed binding site of gH-gL complex. In all the compounds analyzed, O atom on C = O and -OH functional groups were observed to play a key role in hydrogen bonds formation. Figures 5 and 6 depicts the interactions of the 6 best compounds analyzed towards the target binging site of gH-gL complex that displayed good pharmacological properties with no toxicity.

**Table 1:** The binding free energy docking simulation result of ethno compounds toward gH-gL complex of HSV and the number of hydrogen bonds formed along with the amino acid residues involved in the interactions

**S. No**	**Name of the compound**	**Binding energy in K.cal/mol**	**No. of H-Bonds formed with receptor**	**Amino acid residues involved in H-bonding**
1	Aloe-emodin	-9.6	5	ARG 318, TYR 319, SER 349 and ALA 392
2	Allicin	-7.8	2	TYR 319 and ASN 395
3	Anthra quinone	-9.0	1	HIS 398
4	Apigenin	-7.6	2	PRO 346 and ASN 395
5	Baicalein	-7.9	3	ASN 395, PRO 346 and PRO 186
6	Bisbenzylisoquinoline	-10.1	2	ASN 395 and TYR 319
7	Caffeic acid	-7.5	3	ARG 318, HIS 398 and THR 251
8	Caffeoylquinic acid	-8.9	8	THR 251, ARG 318, TYR 319, ASN 395 and HIS 398
9	Catechin	-8.0	4	ARG 318, GLY 270 and THR 350
10	Cochinolide	-9.0	5	ARG 318, TYR 319 and ASN 395
11	Epiafzelechin	-8.3	2	GLY 268 and VAL 394
12	Galangin	-8.3	1	PRO 186
13	Isoborneol	-6.4	0	NIL
14	Kaempferol	-7.9	5	ARG 318, ASN 395 and ALA 392
15	Lignan	NALP	0	NIL
16	Linalool	-7.3	0	NIL
17	(3-chloro phenyl) methyl-3,4,5 trihydroxybenzoate	-10.4	4	ARG 318, TYR 319, ASP 187 and ASN 395
18	Methylgallate	-8.4	4	THR 251, GLY 270 and HIS 398
19	Morin	-8.1	2	ASN 395 and SER 349
20	Oxyresveratrol	-7.2	1	THR 251
21	Phloroglucinol	-7.5	2	GLY 268 and HIS 398
22	Physcion	-8.9	7	ARG 318, ASN 395, PRO 346, SER 349 and ASP 187
23	Quercetin	-8.0	5	ARG 318, GLY 270 and ALA 392
24	Resveratrol	-9.5	2	GLY 268 and HIS 398
25	Rhein	-8.9	3	ARG 318 and ASP 187
26	Rosmarinic acid	-10.1	4	GLY 270, ARG 318 and PRO 346
27	Wogonin	-9.3	5	ARG 318, TYR 319 and ASN 395
28	Yatein	-7.6	1	ARG 318
29	Zeatin	-7.8	4	ARG 318, TYR 319 and HIS 398

NALP = No acceptable ligand poses.

### Binding free energy (ΔG binding)

**Figure 7:**
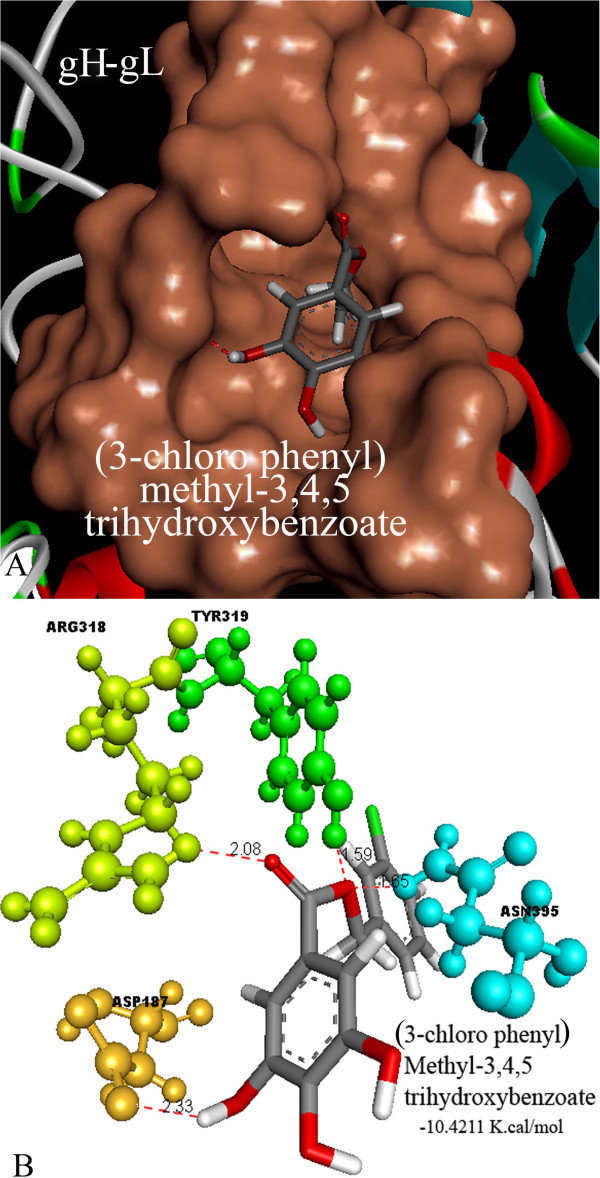
**Docking simulation result of Best binder. A**) Binding of (3-chloro phenyl) methyl-3, 4, 5 trihydroxybenzoate into the proposed binding site of gH-gL complex. **B**) Amino acid residues ARG 318, TYR 319, ASP 187 and ASN 395, which are involved in the binding of the compound in the proposed binding site of gH-gL complex by forming hydrogen bonds (red dotted lines) using -10.4 K.cal./mol of binding energy.

As per the docking results (Table 1), all the 29 compounds tested docked into the active site of the target protein molecule successfully, with a binding energy ranging between -10.4 to -6.4 K.cal./mol. Out of all the compounds analyzed, (3-chloro phenyl) methyl-3,4,5 trihydroxybenzoate showed the least possible binding energy of -10.4 K.cal./mol by forming 4 hydrogen bonds with amino acid residues ASP 187, ARG 318, TYR 319 and ASN 395 (Figure 7). Whereas, Isoborneol and linalool showed the highest possible free binding energy of -6.4 K.cal./mol. and -7.3 K.cal./mol respectively, with no hydrogen bond formation with any of the active site amino acid residues. Caffeoylquinic acid formed the highest possible hydrogen bonds (8 bonds) with THR 251, ARG 318, TYR 319, ASN 395 and HIS 398 amino acid residues of the target protein receptor with a binding energy of -8.9 K.cal./mol. Whereas Lignan was unable to dock into the proposed binding site.

The argusdock values of ΔG binding given in Table 1 shows that every ligand has negative ΔG, which shows that all the ethno medicinal compounds analyzed for binding with gH-gL complex are highly stable. These negative binding free energies also evidenced that the reaction is spontaneous.

### Prediction of pharmacological properties

Lipinski Filter and Osiris Property Explorer were utilized to screen the drug candidate based on Lipinski’s Rule of Five and Oral Bioavailability. The pharmacological attributes prediction results are displayed in (Additional file 1: Table S1) and the structures of all the ethno medicinal compounds used for the study are shown in Additional file 1: Figure S1.

Based on the experimental values, it was inferred that all the ethno medicinal compounds successfully satisfied all the parameters of Lipinski’s Rule of Five with the exception of the hydrogen donor for Caffeoylquinic acid. The parameters of the Lipinski’s rule are as follows: the molecular weight must be < 500 Da, Log P < 5, the number of hydrogen donors must be < 5, the number of acceptor hydrogens must be < 10, and the refractivity molar range must be between 40–130.

As per the veber’s rule, oral bioavailability of drugs could be measured by the molecular weight, number of rotatable bonds (n rotb), number of hydrogen bonds, and the expanse of the drug’s polar surface (TPSA). The oral bioavailability was marked by small molecular weight (less than 500 Da); also, the number of rotatable bond must be less than 10, the number of hydrogen bond donors and acceptors must be less than 12, and TPSA values less than 140. Additional file 1: Table S1 shows that all the compounds have a good oral bioavaibility, with the exception of Caffeoylquinic acid and Rosmarinic acid.

The hydrophobicity of drugs could be inferred from Log P value. Log P Values are directly proportional to the oral hydrophobicity of the drug. More hydrophobic the drug is, higher is the ability of the drug to circulate longer in our body. It wouldn’t be easy to excrete such a drug. In the present investigation, the Log P values of the drug molecules were observed to be in the range of -0.4 to 6. The highest Log P value of 6 was observed for Bisbenzylisoquinoline, followed by 3.8 for Lignan and Yatein, which shows that Bis-benzylisoquinoline was the most hydrophobic molecule amongst all the analyzed. However, Anthraquinone, Galangin, Isoborneol, Lignan, Linalool, (3-chloro phenyl) methyl-3,4,5 trihydroxybenzoate, Oxyresveratrol, Physcion, Resveratrol, Rhein, Rosmarinic acid, Wogonin and Yatein also showed good hydrophobicity with a range of log P between 2–5.

The drug score value is one such useful practical value which is a combination of variables such as drug likeness, Log P, solubility, molecular weight and toxicity risk, which could be used for the evaluation of a potential drug candidate. A drug molecule with a better drug score has a greater chance to be a drug candidate. As per the values shown in Additional file 1: Table S1, Catechin with a drug score 0.87 showed the highest possibility of being a potential drug and Zeatin with a drug score of 0.1 shown to be the least possible potential drug.

### Prediction of toxicity

Osiris Property Explorer and Lazar were used to predict toxicity of the compounds in the present study. The predictions were based on the functional group similarity for the query molecule with the *in vitro* and *in vivo* validated compounds present in the database of these two online programs.

Osiris Property Explorer results were visualized using color codes. The result of toxicity analysis of all the analyzed ethno compounds is shown in Additional file 1: Table S2. Green color shows low toxic tendency, yellow shows the midcore and red color shows high tendency of toxicity.

Additional file 1: Table S2 shows that Anthraquinone, Apigenin, Caffeic acid, Galangin, Kaempferol, Linalool, Morin, Phloroglucinol, Quercetin, Resveratrol, Zeatin have high toxicity. Whereas Allicin, Bis-benzylisoquinoline, Caffeoylquinic acid, Catechin, Cochinolide, Epiafzelechin, Isoborneol, Lignan, (3-chloro phenyl) methyl-3,4,5 trihydroxybenzoate, Methylgallate, Oxyresveratrol, Rhein, Rosmarinic acid, Wogonin and Yatein had no toxic effect, whereas other compounds had midcore toxicity.

Lazar is a software package which is used to detect mutagenic and/or carcinogenic properties based on the similarities in functional group with mutagenic and/or carcinogenic ones present in its database. Additional file 1: Table S3 shows the results of compounds toxicity analysis by lazar. The training dataset of molecules present in Lazar program were verified by conducting an assay with *Salmonella typhimurium*.

Additional file 1: Table S3 shows that Aloe-emdoin, Anthraquinone, Baicalein, Bisbenzylisoquinoline, Galangin, Morin, Physcion, Quercetin, Rhein, Wogonin and Zeatin have mutagenic properties. Whereas Caffeic acid, Caffeoylquinic acid, Catechin, Cochinolide, Epiafzelechin, Lignan, Linalool, (3-chloro phenyl) methyl-3,4,5 trihydroxybenzoate, Methylgallate, Oxyresveratrol, Phloroglucinol, Resveratrol, Rosmarinic acid and Yatein have no mutagenic properties. Moreover, the carcinogenicity of compounds was verified by *in silico* animal testing studies with Mouse. Additional file 1: Table S3 shows that all the compounds analyzed have no carcinogenicity, with the exception of Zeatin.

### Screening for the best compound based on docking and drug likeliness results

It was found that (3-chloro phenyl) methyl-3,4,5 trihydroxybenzoate was the best anti herpes viral ethno medicinal compound based on its ΔG binding compared to other compounds followed by Bis-benzylisoquinoline and Rosmarinic acid with a binding energy of around -10 K.cal/mol. However, Bis-benzylisoquinoline and Rosmarinic acid have bad pharmacological and toxicity attributes, based on pharmacological properties. Rosmarinic acid has a TPSA value more in excess of threshold Veber’s rule parameter, whereas Bisbenzylisoquinoline showed considerable mutagenicity. (3-chloro phenyl) methyl-3,4,5 trihydroxybenzoate showed good pharmacological attributes, since it satisfied the Lipinski’s Rule, Veber’s Rule, Log P values, toxicity and drug score threshold.

However, based on Pharmacological properties, Caffeoylquinic acid, Catechin, Cochinolide, Epiafzelechin, Lignan, (3-chloro phenyl) methyl-3,4,5 trihydroxybenzoate, Methylgallate, Oxyresveratrol, Rosmarinic acid and Yatein showed good pharmacological attributes. These compounds were found to comply with Lipinski’s rule, Veber’s rule and oral bioavailability parameters with the exception of Caffeoylquinic acid and Rosmarinic acid, because of their TPSA value exceeded the threshold of 140.

## Conclusion

The binding energy analysis showed that (3-chloro phenyl) methyl-3,4,5 trihydroxybenzoate had a least binding energy value of -10.4 K.cal/mol compared to other compounds followed by Bisbenzylisoquinoline and Rosmarinic acid with binding energy values of -10.1 K.cal/mol each, among all the compounds analyzed. Visualization of the interactions showed that O atom on C = O and -OH groups of all tested compounds played a major role in hydrogen bonds formation with amino acid residues of receptor molecule. Conclusively, our results strongly suggest that (3-chloro phenyl) methyl-3, 4, 5 trihydroxybenzoate is worth a lead ethno compound for developing further as a potent inhibitor against Herpes simplex virus targeting its fusion protein complex. On the other hand, successful repositioning of the analyzed compounds onto the proposed binding site of gH-gL complex shed light on its drug targetability for further investigations. However, as this present work is only a step forward towards understanding the mechanistic insights of a potent inhibitor for herpes viral entry into the host cell targeting gB-gH-gL complex interface, further *in vitro* and *in vivo* validations are required.

## Competing interests

The authors declare that they have no competing interests.

## Authors’ contributions

SHB conceptualized the idea, carried out the work including acquisition of the data, analysis and interpretation of the final results and drafting the manuscript. TD assisted SHB in acquisition of the data and RNP helped with editing the manuscript. All the authors have read and approved the final manuscript.

## Pre-publication history

The pre-publication history for this paper can be accessed here:

http://www.biomedcentral.com/1472-6882/13/85/prepub

## Supplementary Material

Additional file 1: Table S1 The molecular descriptor values of the ethno compounds used in this study along with their pubchem compound IDs. **Table S2** Toxicity of ethno compounds based on Osiris Property Explorer predictions. **Table S3** Toxicity of ethno compounds based on Lazar program predictions **Figure S1** Structures of the ethno compounds used in the present study. Click here for file
