# Supplementary material for: Computational repositioning of ethno medicine elucidated gB-gH-gL complex as novel anti herpes drug target
Source: BMC Complement Altern Med. 2013 Apr 15;13:85. doi: 10.1186/1472-6882-13-85 (PMC3662606; doi:10.1186/1472-6882-13-85)
Supplement: Additional file 1: Table S1 — The molecular descriptor values of the ethno compounds used in this study along with their pubchem compound IDs. Table S2 Toxicity of ethno compounds based on Osiris Property Explorer predictions. Table S3 Toxicity of ethno compounds based on Lazar program predictions Figure S1 Structures of the ethno compounds used in the present study. [file 1472-6882-13-85-S1.docx]

**Table S1:** The molecular descriptor values of the ethno compounds used in this study along with their pubchem compound IDs.

| **S.No** | **Name of the compound** | **Pubchem Compound ID** | **Molecular Formula** | **Mol. wt.** | **Log P** | **No. of H-bond donors** | **No. of H-bond acceptors** | **No. of rotatable bonds** | **TPSA** | **Drug score** |
| --- | --- | --- | --- | --- | --- | --- | --- | --- | --- | --- |
|  | Aloe-emodin | 10207 | C_15_H_10_O_5_ | 270.23 | 1.8 | 3 | 5 | 1 | 101.15 | 0.34 |
|  | Allicin | 65036 | C_6_H_10_OS_2_ | 162.27 | 1.3 | 0 | 1 | 5 | 61.6 | 0.48 |
|  | Anthra quinine | 6780 | C_14_H_8_O_2_ | 208.21 | 3.4 | 0 | 2 | 0 | 34.14 | 0.08 |
|  | Apigenin | 5280443 | C_15_H_10_O_5_ | 270.23 | 1.7 | 3 | 5 | 1 | 87.0 | 0.22 |
|  | Baicalein | 5281605 | C_15_H_10_O_5_ | 270.23 | 1.7 | 3 | 5 | 1 | 87.0 | 0.44 |
|  | Bisbenzylisoquinoline | 22169421 | C_23_H_19_N | 309.40 | 6 | 0 | 1 | 4 | 12.890 | 0.24 |
|  | Caffeic acid | 689043 | C_9_H_8_O_4_ | 180.15 | 1.2 | 3 | 4 | 2 | 77.76 | 0.19 |
|  | Caffeoylquinic acid | 1794427 | C_16_H_18_O_9_ | 354.30 | -0.4 | 6 | 9 | 5 | 164.75 | 0.62 |
|  | Catechin | 9064 | C_15_H_14_O_6_ | 290.26 | 0.4 | 5 | 6 | 1 | 110.38 | 0.87 |
|  | Cochinolide | 10332730 | C_15_H_14_O_4_ | 258.26 | 1.3 | 2 | 4 | 2 | 66.76 | 0.79 |
|  | Epiafzelechin | 443639 | C_15_H_14_O_5_ | 274.26 | 0.7 | 4 | 5 | 1 | 90.15 | 0.85 |
|  | Galangin | 5281616 | C_15_H_10_O_5_ | 270.23 | 2.3 | 3 | 5 | 1 | 87.0 | 0.28 |
|  | Isoborneol | 64685 | C_10_H_18_O | 154.24 | 2.7 | 1 | 1 | 0 | 20.23 | 0.48 |
|  | Kaempferol | 5280863 | C_15_H_10_O_6_ | 286.23 | 1.9 | 4 | 6 | 1 | 107 | 0.22 |
|  | Lignan | 261166 | C_25_H_30_O_8_ | 458.50 | 3.8 | 0 | 8 | 9 | 89.52 | 0.6 |
|  | Linalool | 6549 | C_10_H_18_O | 154.24 | 2.7 | 1 | 1 | 4 | 20.23 | 0.13 |
|  | (3-chloro phenyl) methyl-3,4,5 trihydroxybenzoate | 10732589 | C_14_H_11_ClO_5_ | 294.68 | 2.8 | 3 | 5 | 4 | 86.99 | 0.72 |
|  | Methylgallate | 7428 | C_8_H_8_O_5_ | 184.14 | 0.9 | 3 | 5 | 2 | 86.99 | 0.55 |
|  | Morin | 5281670 | C_15_H_10_O_7_ | 302.23 | 1.5 | 5 | 7 | 1 | 127 | 0.28 |
|  | Oxyresveratrol | 5281717 | C_14_H_12_O_4_ | 244.24 | 2.8 | 4 | 4 | 2 | 80.92 | 0.46 |
|  | Phloroglucinol | 359 | C_6_H_6_O_3_ | 126.11 | 0.2 | 3 | 3 | 0 | 60.69 | 0.18 |
|  | Physcion | 10639 | C_16_H_12_O_5_ | 284.26 | 3 | 2 | 5 | 1 | 83.83 | 0.33 |
|  | Quercetin | 5280343 | C_15_H_10_O_7_ | 302.23 | 1.5 | 5 | 7 | 1 | 127 | 0.32 |
|  | Resveratrol | 445154 | C_14_H_12_O_3_ | 228.24 | 3.1 | 3 | 3 | 2 | 60.69 | 0.22 |
|  | Rhein | 10168 | C_15_H_8_O_6_ | 284.22 | 2.2 | 3 | 6 | 1 | 111.90 | 0.61 |
|  | Rosmarinic acid | 5281792 | C_18_H_16_O_8_ | 360.31 | 2.4 | 5 | 8 | 7 | 144.52 | 0.49 |
|  | Wogonin | 5281703 | C_16_H_12_O_5_ | 284.26 | 3 | 2 | 5 | 2 | 76.0 | 0.43 |
|  | Yatein | 442835 | C_22_H_24_O_7_ | 400.42 | 3.8 | 0 | 7 | 7 | 72.45 | 0.69 |
|  | Zeatin | 449093 | C_10_H_13_N_5_O | 219.24 | 0.7 | 3 | 1 | 4 | 86.72 | 0.1 |

**Table S2:** Toxicity of ethno compounds based on Osiris Property Explorer predictions:

| **S.No** | **Name of the compound** | **Mutagenic** | **Tumorigenic** | **Irritant** | **Reproductive effective** |
| --- | --- | --- | --- | --- | --- |
|  | Aloe-emodin | Red | Green | Green | Green |
|  | Allicin | Green | Green | Green | Green |
|  | Anthra quinone | Red | Red | Red | Green |
|  | Apigenin | Red | Yellow | Green | Red |
|  | Baicalein | Red | Green | Green | Green |
|  | Bisbenzylisoquinoline | Green | Green | Green | Green |
|  | Caffeic acid | Red | Red | Green | Red |
|  | Caffeoylquinic acid | Green | Green | Green | Green |
|  | Catechin | Green | Green | Green | Green |
|  | Cochinolide | Green | Green | Green | Green |
|  | Epiafzelechin | Green | Green | Green | Green |
|  | Galangin | Red | Yellow | Green | Yellow |
|  | Isoborneol | Green | Green | Green | Green |
|  | Kaempferol | Red | Yellow | Green | Red |
|  | Lignan | Green | Green | Green | Green |
|  | Linalool | Red | Green | Red | Yellow |
|  | (3-chloro phenyl) methyl-3,4,5 trihydroxybenzoate | Green | Green | Green | Green |
|  | Methylgallate | Green | Green | Green | Green |
|  | Morin | Red | Yellow | Green | Yellow |
|  | Oxyresveratrol | Green | Green | Green | Green |
|  | Phloroglucinol | Red | Green | Green | Red |
|  | Physcion | Yellow | Green | Green | Green |
|  | Quercetin | Red | Yellow | Green | Yellow |
|  | Resveratrol | Yellow | Green | Green | Red |
|  | Rhein | Green | Green | Green | Green |
|  | Rosmarinic acid | Green | Green | Green | Green |
|  | Wogonin | Green | Green | Green | Green |
|  | Yatein | Green | Green | Green | Green |
|  | Zeatin | Red | Green | Red | Red |

**Table S3:** Toxicity of ethno compounds based on Lazar program predictions:

|  |  | **Mutagenicity** | | **Carcinogenicity** |
| --- | --- | --- | --- | --- |
| **S.No** | **Name of the compound** | **Salmonella typhimurium (CPDB)** | **Salmonella typhimurium (Kazius/Bursi)** | **Mouse** |
|  | Aloe-emodin | Yes | Yes | No |
|  | Allicin | Yes | No | No |
|  | Anthra quinone | Yes | Yes | NA |
|  | Apigenin | Yes | No | No |
|  | Baicalein | Yes | Yes | No |
|  | Bisbenzylisoquinoline | Yes | Yes | NA |
|  | Caffeic acid | No | No | No |
|  | Caffeoylquinic acid | No | No | No |
|  | Catechin | No | No | No |
|  | Cochinolide | No | No | No |
|  | Epiafzelechin | No | No | No |
|  | Galangin | Yes | Yes | No |
|  | Isoborneol | No | NA* | No |
|  | Kaempferol | Yes | No | No |
|  | Lignan | No | No | No |
|  | Linalool | No | No | No |
|  | (3-chloro phenyl) methyl-3,4,5 trihydroxybenzoate | No | No | No |
|  | Methylgallate | No | No | No |
|  | Morin | Yes | Yes | No |
| 1. e | Oxyresveratrol | No | No | No |
|  | Phloroglucinol | No | No | No |
|  | Physcion | Yes | Yes | No |
|  | Quercetin | Yes | Yes | No |
|  | Resveratrol | No | No | No |
|  | Rhein | Yes | Yes | No |
|  | Rosmarinic acid | No | No | No |
|  | Wogonin | Yes | Yes | No |
|  | Yatein | No | No | No |
| 1. n | Zeatin | Yes | Yes | Yes |

NA = Not enough similar compounds in training dataset. NA*= No measured activity

**
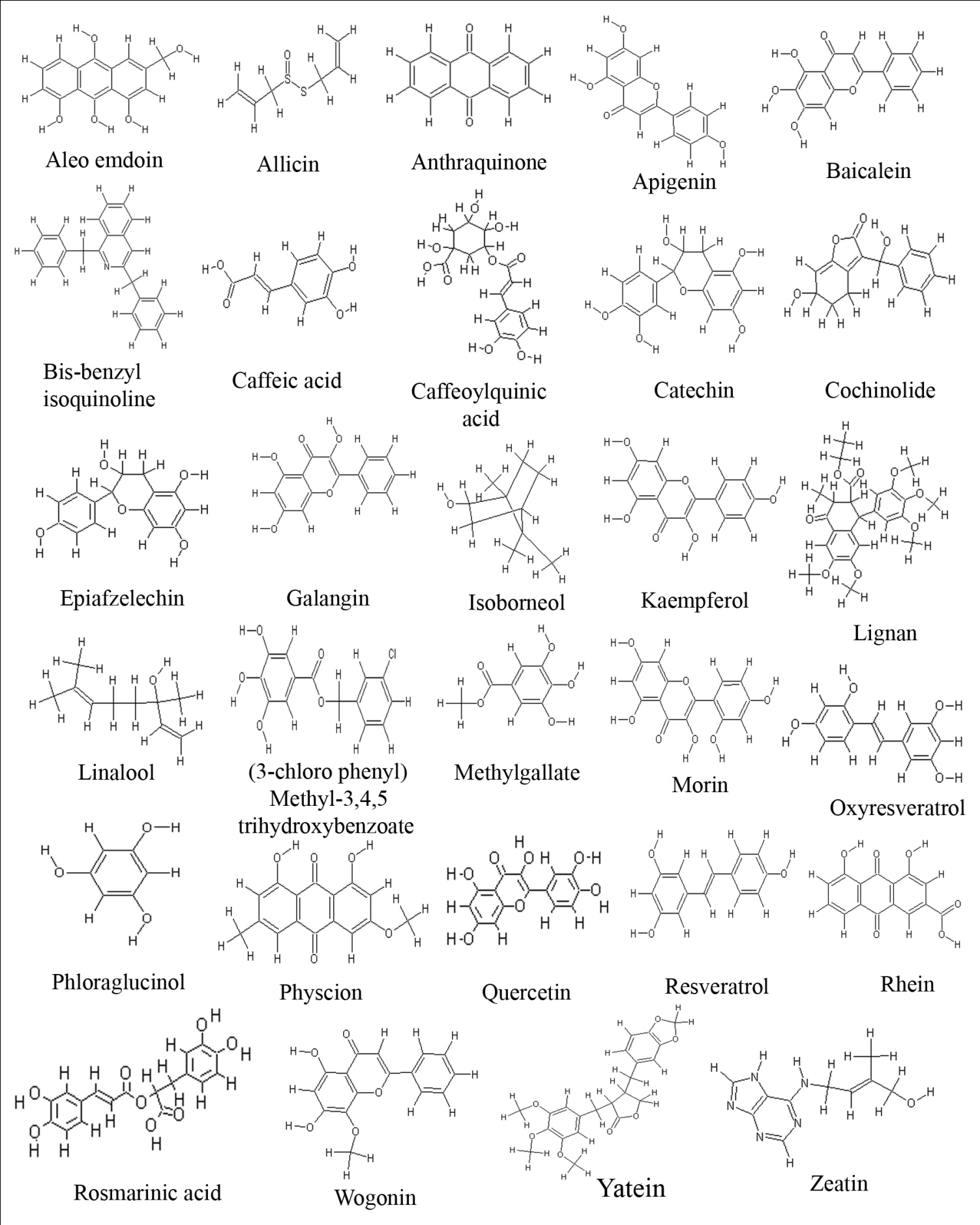
Figure S1:** Structures of the ethno compounds used in the present study.
